# Predictors for development of palbociclib-induced neutropenia in breast cancer patients as determined by ordered logistic regression analysis

**DOI:** 10.1038/s41598-021-99504-5

**Published:** 2021-10-08

**Authors:** Yuko Kanbayashi, Koichi Sakaguchi, Takeshi Ishikawa, Koichi Takayama, Tetsuya Taguchi

**Affiliations:** 1 Department of Outpatient Oncology Unit, University Hospital, Kyoto Prefectural University of Medicine, Kyoto, Japan; 2 Department of Education and Research Center for Clinical Pharmacy, Faculty of Pharmacy, Osaka Medical and Pharmaceutical University, 4-20-1 Nasahara, Takatsuki, Osaka 569-1094 Japan; 3 Department of Endocrine and Breast Surgery, Graduate School of Medical Science, Kyoto Prefectural University of Medicine, Kyoto, Japan; 4 Department of Molecular Gastroenterology and Hepatology, Graduate School of Medical Science, Kyoto Prefectural University of Medicine, Kyoto, Japan; 5 Department of Pulmonary Medicine, Graduate School of Medical Science, Kyoto Prefectural University of Medicine, Kyoto, Japan

**Keywords:** Endocrinology, Health care, Medical research, Oncology, Risk factors

## Abstract

This retrospective study aimed to identify predictors for the development of palbociclib-induced neutropenia. This study retrospectively analysed 78 breast cancer patients who had received palbociclib at our hospital between January 2018 and May 2020. For the regression analysis of factors associated with palbociclib-induced neutropenia, variables were extracted manually from medical charts. The level of palbociclib-induced neutropenia was evaluated using the National Cancer Institute’s Common Terminology Criteria for Adverse Events (version 5). Multivariate ordered logistic regression analysis was performed to identify predictors for the development of neutropenia. Optimal cut-off thresholds were determined using receiver operating characteristic (ROC) analysis. Values of *P* < 0.05 (2-tailed) were considered significant. Significant factors identified included concomitant use of statin (odds ratio [OR] = 0.104, 95% confidence interval [CI] = 0.018–0.598; *P* = 0.011) and body mass index (BMI) (OR = 1.118, 95% CI = 1.007–1.241; *P* = 0.037). ROC analysis revealed that neutropenia (grade 4) was more likely to occur with a BMI ≥ 22.3 kg/m^2^. In conclusion, no concomitant use of statins and high BMI were identified as significant predictors for the development of palbociclib-induced neutropenia.

## Introduction

Palbociclib plus endocrine therapy is the standard treatment for hormone receptor-positive, human epidermal growth factor receptor 2-negative metastatic breast cancer^1–9^. On the other hand, severe neutropenia has been reported as an adverse effect after palbociclib administration^6,9^. Neutropenia is the dose-limiting toxicity associated with palbociclib. In some clinical trials, it has been reported that the primary toxicity of asymptomatic neutropenia was effectively managed by dose modification without apparent loss of efficacy^7^. However, in daily clinical practice, occasionally, palbociclib was permanently discontinued due to grade 4 neutropenia. The development of neutropenia poses a risk of febrile neutropenia and may be associated with shortened life prognosis due to discontinuation^10^, leading to decreases in quality of life (QOL).

This retrospective study was thus undertaken to identify predictors associated with the development of palbociclib-induced neutropenia to help guide future strategies toward improved safety, efficacy, and QOL in breast cancer patients treated using palbociclib.

## Results

All 78 patients who received palbociclib were enrolled in this study. Table 1 presents the clinical characteristics of the 78 enrolled patients, the potential variables related to the development of neutropenia, and the results of univariate analyses. The forward stepwise selection procedure identified the following candidate variables: use of statins, use of proton pump inhibitors (PPIs), number of cycles, body mass index (BMI) and platelet count. Multivariate ordered logistic regression analysis was performed using these variables. Significant factors identified included concomitant use of statin (odds ratio [OR] = 0.104, 95% confidence interval [CI] = 0.018–0.598; *P* = 0.011) and BMI (OR = 1.118, 95% CI = 1.007–1.241; *P* = 0.037) (Table 2). Receiver operating characteristic (ROC) analysis revealed that neutropenia (grade 4) was more likely to occur with BMI ≥ 22.3 kg/m^2^, with 71.0% sensitivity and 48.4% specificity (area under the curve [AUC] = 0.54).

**Table 1:** Patient characteristics, extracted variables, and results of univariate analyses (n = 78).

	Grade 1 (n = 4)	Grade 2 (n = 15)	Grade 3 (n = 45)	Grade 4 (n = 14)	*P* value	Odds ratio (95%CI)
**Demographic data**						
Age (y), median (range)	73.5 (58–77)	60 (39–73)	61 (40–83)	59.5 (46–79)	0.710	0.99 (0.96–1.03)
Height (cm), median (range)	157 (144–161)	152 (145–68)	156 (144–167)	156 (148–165)	0.111	1.06 (0.99–1.14)
Weight (kg), median (range)	43.6 (31.6–62.0)	50 (40–67)	54 (40–85)	55.5 (33.6–78.9)	0.097	1.04 (0.99–1.08)
BMI (kg/m^2^), median (range)	17.8 (14.9–23.9)	21.0 (17.3–29.7)	22.5 (16.6–35.8)	21.0 (15.3–34.9)	0.310	1.05 (0.95–1.16)
BMI ≥ 25 kg/m^2^, n (%)	0	4 (26.7)	15 (33.3)	4 (28.5)	0.51	1.38 (0.53–3.57)
PS (0/1/2/3)	3/0/1/0	9/5/1/0	23/19/3/0	8/4/1/1	0.452	1.27 (0.68–2.38)
**Comorbidities**						
Diabetes mellitus, n (%)	0	3 (20.0)	4 (8.9)	2(14.3)	0.878	0.90 (0.23–3.46)
Hyperlipidaemia, n (%)	1 (25.0)	4 (26.7)	7 (15.6)	0	0.056	0.31 (0.09–1.03)
**Laboratory test values before administration**						
Serum creatinine, mg/dL, median (range)	0.62 (0.57–0.65)	0.62 (0.41–1.19)	0.68 (0.38–1.37)	0.58 (0.36–0.8)	0.541	0.49 (0.05–4.93)
Creatinine clearance, mL/min, median (range)	58.1 (37.3–92.3)	81.0 (34.2–118.9)	80.0 (26.9–188.4)	89.9 (33.9–128.1)	0.149	1.01 (1.00–1.03)
Albumin, g/dL, median (range)	3.9 (3.4–4.2)	3.9 (3.3–4.8)	4.0 (2.3–4.8)	3.9 (2.9–5)	0.908	1.06 (0.40–2.79)
Alanine aminotransferase, U/L, median (range)	14.5 (13–20)	19 (8–62)	16 (6–142)	20 (6–106)	0.504	1.01 (0.99–1.03)
Total bilirubin, mg/dL	0.72 (0.45–1.11)	0.49 (0.38–0.81)	0.72 (0.27–2.2)	0.74 (0.36–1.27)	0.117	2.92 (0.76–11.1)
Neutrophils, × 10^3^/μL, median (range)	5.46 (2.35–7.53)	2.42 (0.82–5.48)	3.28 (0.53–7.41)	2.23 (0.82–9.98)	0.349	0.89 (0.69–1.14)
Platelets, × 10^3^/μL, median (range)	313 (223–350)	263 (144–431)	241 (116–380)	224 (83–457)	0.037*	0.994 (0.988–0.999)
Haemoglobin, g/dL, median (range)	12.1 (10.7–13.9)	11.9 (8.7–14.4)	12.9 (9.0–15.6)	12.4 (9.6–14.7)	0.368	1.14 (0.85–1.53)
Number of cycles, median (range)	7.5 (1–15)	10.5 (2–44)	7 (1–37)	8.5 (1–38)	0.302	0.98 (0.94–1.02)
**Concomitant medications**						
Statin, n (%)	1 (25)	2 (13.3)	3 (6.7)	0	0.077	0.24 (0.05–1.17)
Proton pump inhibitor, n (%)	0	3 (20.0)	6 (13.3)	0	0.289	0.49 (0.13–1.85)
**Sites of metastatic disease, n (%)**						
Bone, n (%)	2(50)	10 (66.7)	30 (66.7)	10 (71.4)	0.581	1.29 (0.52–3.23)
Liver, n (%)	1 (25)	3 (20)	18 (40)	6 (42.9)	0.173	1.89 (0.76–4.75)
Lung, n (%)	2 (50)	5 (33.3)	13 (28.9)	6 (42.9)	0.872	1.08 (0.43–2.69)
Skin, n (%)	0	1 (6.7)	4 (8.9)	1 (7.1)	0.774	1.27 (0.25–6.44)
Brain, n (%)	0	1 (6.7)	2 (4.4)	0	0.563	0.52 (0.06–4.71)
Others, n (%)	1 (25)	1 (6.7)	4 (8.9)	1 (7.1)	0.640	0.70 (0.16–3.13)
Prior lines of chemotherapy in metastatic disease, median (range)	0.5 (0–5)	2 (0–6)	2 (0–8)	2 (0–10)	0.489	1.07 (0.89–1.29)
Prior lines of ET in metastatic disease, median (range)	2.5 (1–4)	2 (0–5)	1(0–6)	2 (0–4)	0.326	0.86 (0.63–1.17)
**Prior ET for advanced disease**						
Tamoxifen, n (%)	0	5 (33.3)	18 (40)	3 (21.4)	0.968	1.02 (0.41–2.54)
Fulvestrant, n (%)	1(25)	4(26.7)	16 (35.6)	4 (28.6)	0.803	1.12 (0.45–2.84)
Aromatase inhibitor, n (%)	4 (100)	11(73.3)	25 (55.6)	9 (64.3)	0.258	0.59 (0.24–1.47)
**Palbociclib endocrine partner**						
Aromatase inhibitor, n (%)	4 (100)	4 (26.7)	14 (31.1)	7 (50)	0.99	1.01 (0.41–2.45)
Fulvestrant, n (%)	0	11(73.3)	31 (68.9)	7 (50)	0.99	0.99 (0.41–2.43)

CI, confidence interval; BMI, body mass index; PS, ECOG performance status; ET, Endocrine therapy.

*P < 0.05.

**Table 2:** Results of multivariate ordered logistic regression analysis for variables extracted by forward selection (n = 78).

Variable	*P* value	Odds ratio	95%CI	
			Lower endpoint	Upper endpoint
Statin	0.011*	0.104	0.018	0.598
Proton pump inhibitor	0.091	0.290	0.069	1.220
Number of cycles	0.130	0.966	0.924	1.010
BMI	0.037*	1.118	1.007	1.241
Platelets	0.076	0.995	0.989	1.001

CI, confidence interval; BMI, body mass index.

*P < 0.05.

## Discussion

The multivariate ordered logistic regression analysis performed in this study showed that significant predictors for the development of palbociclib-induced neutropenia included use of statins and BMI. Platelet count at the start of palbociclib was not extracted as a significant variable in multivariate analysis, but the lower the platelet count at the start of palbociclib administration, the more likely neutropenia was to develop. PPI was also not extracted as a significant variable, although combined use of PPI tended to be associated with neutropenia.

When the body fat content is high, anticancer drugs reportedly dissolve in adipose tissue and excretion is thus delayed. In this study, BMI was extracted as a significant predictor for the development of palbociclib-induced neutropenia. This is consistent with the results of previous studies^11,12^. On the other hand, there are many reports contrary to the results of this study that the effect of anticancer drugs is diminished in obese patients^13–16^. Further verification is needed in this issue.

The ROC curve analysis revealed a BMI cut-off of ≥ 22.3 kg/m^2^ for the group likely to develop neutropenia (Grade 4). World Health Organization have defined obesity or overweight patients as individuals with BMI ≥ 25 kg/m^2^^17^. However, in this study, neutropenia was more likely to occur with BMI ≥ 22.3 kg/m^2^. This might be due to the fact that Asians were more likely to develop neutropenia than non-Asians^18^. Clinicians might be better to pay close attention to the onset of neutropenia after palbociclib administration among patients with BMI ≥ 22.3 kg/m^2^.

As for concomitant pharmacotherapies, statin use was extracted as a significant factor in this study. Concomitant use of statins resulted in a lower risk of neutropenia. This result is consistent with the results of basic research suggesting that statins prevent leukopenia^19^. Hypercholesterolemia has been reported as a risk factor for breast cancer^20^, and statins are used for patients with hypercholesterolemia. Statins have also been reported to reduce the risk of breast cancer^21^. The concomitant use of statins and palbociclib might be useful in the treatment of breast cancer. However, there are many reports that statins do not reduce the risk of breast cancer^22^. Further research is needed in this issue.

Although not significant, concomitant use of PPIs was suggested to potentially diminish the effect of palbociclib. Previous research has reported that co-administration of PPIs was significantly related to the development of neutropenia^23^. Regarding the relationship between combined use of PPIs and the occurrence of neutropenia, an increase in gastric pH due to the inhibitory effects of PPIs on gastric acid secretion may reduce the absorption of palbociclib^24^.

Furthermore, although not significant, low pretreatment platelet count was also suggested as a risk factor for neutropenia. This result is consistent with previous findings^25,26^. Clinicians thus need to know about the incidence and severity of neutropenia, especially in patients with low platelet counts before palbociclib administration.

Several limitations to the current study need to be considered. First, the retrospective nature of the study may have decreased the validity of the data obtained. Second, since this study was performed at a single institute, prospective multicentre studies are needed to confirm the results.

In conclusion, concomitant use of statins and BMI were identified as significant predictors for the development of palbociclib-induced neutropenia in breast cancer. However, our findings need to be confirmed in further studies. Nevertheless, these results may assist in developing strategies to improve the safety, efficacy, and QOL among patients receiving palbociclib.

## Patients and methods

### Study period and participants

This study retrospectively analysed 78 female breast cancer patients who had receiving palbociclib at our hospital between January 2018 and May 2020. The Medical Ethics Review Committee of the Kyoto Prefectural University of Medicine approved this study (approval no. ERB-C-1837–1). All procedures were performed in accordance with the ethical standards of the Kyoto Prefectural University of Medicine Institutional Medical Ethics Review Committee and the 1964 Declaration of Helsinki and its later amendments. No prospective studies with human participants or animals were performed by any of the authors for this article. Given the retrospective nature of this work, the need to obtain informed consent was waived for the individual participants included in the study, in accordance with the standards of the Kyoto Prefectural University of Medicine Institutional Medical Ethics Review Committee.

### Extraction of variables

For the regression analysis of factors associated with palbociclib-induced neutropenia, variables were extracted manually from medical charts. Evaluated variables included factors that could potentially impact the development of neutropenia: demographic data (age, height, weight, and BMI), Eastern Cooperative Oncology Group Performance Status (ECOG-PS), presence of comorbidities, laboratory test values, number of cycles, concomitant medications (PPIs, statins), sites of metastatic disease, number of prior lines of chemotherapy, number of prior lines of endocrine therapy, prior endocrine therapy and palbociclib endocrine partner.

Creatinine clearance was estimated using the Cockcroft and Gault equation based on serum creatinine, sex, age, and weight. Clinical information was extracted before administration of the first dose of palbociclib. Concomitant medication was defined as administration of another drug for ≥ 2 weeks at the time of evaluation. The level of palbociclib-induced neutropenia was evaluated using the National Cancer Institute’s Common Toxicity Criteria for Adverse Events (NCI-CTCAE; version 5). The degree of neutropenia was evaluated at the time of onset of the most severe neutropenia for patients who developed neutropenia, and as the lowest neutrophil count level within 1 month after final palbociclib administration for patients who did not develop neutropenia.

### Statistical analysis

Independent variables were analysed for multicollinearity (correlation coefficient |r|≥ 0.7), since correlations among variables can lead to unreliable and unstable results of regression analyses. Independent variables were extracted based on the strength of the correlation with the level of palbociclib-induced neutropenia (dependent variable) or clinical significance. First, univariate ordered logistic regression analyses between outcomes and each potential independent variable were performed. Subsequently, a multivariate ordered logistic regression model was constructed by employing the forward–backward stepwise selection procedure with the resulting candidate variables. The model used a variable entry criterion of 0.15 and a variable retention criterion of 0.1. Ordered logistic regression analysis was employed, because the level of neutropenia was evaluated by a graded scale and multiple factors really associated as predictors for the development of palbociclib-induced neutropenia had to be analysed concurrently. Optimal cut off thresholds were determined using ROC curve analysis.

For all statistical analyses, values of *P* < 0.05 (two-tailed) were considered significant. All analyses were performed using JMP version 14.3.0 (SAS Institute, Cary, NC).
